# Expression of inducible nitric oxide synthase, nitrotyrosine, eosinophilic peroxidase, eotaxin-3, and galectin-3 in patients with gastroesophageal reflux disease, eosinophilic esophagitis, and in healthy controls: a semiquantitative image analysis of 3,3′-diaminobenzidine-stained esophageal biopsies

**DOI:** 10.1093/dote/doae034

**Published:** 2024-04-28

**Authors:** John Plate, Mogens Bove, Helen M Larsson, Elisabeth Norder Grusell, Nabanita Chatterjee, Leif E Johansson, Henrik Bergquist

**Affiliations:** Department of Otorhinolaryngology, Head and Neck Surgery, NU-Hospital Group, Region Västra Götaland, Trollhättan, Sweden; Department of Otorhinolaryngology, Head and Neck Surgery, Institute of Clinical Sciences, Sahlgrenska Academy, University of Gothenburg, Gothenburg, Sweden; Department of Otorhinolaryngology, Head and Neck Surgery, NU-Hospital Group, Region Västra Götaland, Trollhättan, Sweden; Department of Otorhinolaryngology, Head and Neck Surgery, Institute of Clinical Sciences, Sahlgrenska Academy, University of Gothenburg, Gothenburg, Sweden; Department of Otorhinolaryngology, Head and Neck Surgery, NU-Hospital Group, Region Västra Götaland, Trollhättan, Sweden; Department of Otorhinolaryngology, Head and Neck Surgery, Institute of Clinical Sciences, Sahlgrenska Academy, University of Gothenburg, Gothenburg, Sweden; Department of Otorhinolaryngology, Head and Neck Surgery, Kungsbacka Hospital, Region Halland, Kungsbacka, Sweden; Core Facilities, Centre for Cellular Imaging (CCI), University of Gothenburg, Gothenburg, Sweden; Department of ENT, Head and Neck Surgery, Skövde County Hospital, Region Västra Götaland, Skövde, Sweden; Department of Otorhinolaryngology, Head and Neck Surgery, Institute of Clinical Sciences, Sahlgrenska Academy, University of Gothenburg, Gothenburg, Sweden

**Keywords:** eosinophilic esophagitis, gastroesophageal reflux disease, image analysis, immunohistochemistry

## Abstract

Eosinophilic esophagitis (EoE) and gastroesophageal reflux disease (GERD) share many histopathological features; therefore, markers for differentiation are of diagnostic interest and may add to the understanding of the underlying mechanisms. The nitrergic system is upregulated in GERD and probably also in EoE. Esophageal biopsies of patients with EoE (*n* = 20), GERD (*n* = 20), and healthy volunteers (HVs) (*n* = 15) were exposed to antibodies against inducible nitric oxide synthase (iNOS), nitrotyrosine, eosinophilic peroxidase, eotaxin-3, and galectin-3. The stained object glasses were randomized, digitized, and blindly analyzed regarding the expression of DAB (3,3′-diaminobenzidine) by a protocol developed in QuPath software. A statistically significant overexpression of iNOS was observed in patients with any of the two inflammatory diseases compared with that in HVs. Eotaxin-3 could differentiate HVs versus inflammatory states. Gastroesophageal reflux patients displayed the highest levels of nitrotyrosine. Neither iNOS nor nitrotyrosine alone were able to differentiate between the two diseases. For that purpose, eosinophil peroxidase was a better candidate, as the mean levels increased stepwise from HVs via GERD to EoE. iNOS and nitrotyrosine are significantly overexpressed in patients with EoE and GERD compared with healthy controls, but only eosinophil peroxidase could differentiate the two types of esophagitis. The implications of the finding of the highest levels of nitrotyrosine among gastroesophageal reflux patients are discussed.

## Graphical Abstract

**Graphical Abstract:**
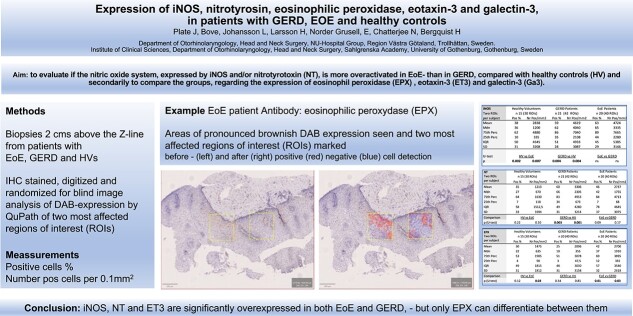

## INTRODUCTION

The most common cause of esophagitis is gastroesophageal reflux disease (GERD), followed by eosinophilic esophagitis (EoE). Histopathologically, the classical signs are shared, while symptoms differ, as esophageal dysfunction is more prominent in EoE.^1^

Nitric oxide (NO) is needed for maintaining normal peristalsis.^2^^,^^3^ In GERD patients, the upregulation of iNOS has been reported to increase mucosal defense.^4^^,^^5^

The esophagus and the lungs share their embryological origin, and expired NO (FeNO) is an important marker for inflammatory activity in asthma, but regarding EoE, the results have been disappointing.^6^ In the esophagus, the expression of iNOS reflects the activation of the NO system.^7^ Another more stable end product of NO is 3-nitrotyrosine (NT), which reflects a more longstanding nitrergic drive.^8^

The purpose of comparing EoE to GERD and to healthy volunteers (HVs) is to search for improved differential diagnostics, but more importantly, to expand our understanding of the underlying mechanisms.

Another proinflammatory marker is galectin-3 (Ga-3), which has been linked with eosinophil recruitment.^9^ Also other eosinophil cell-specific markers, such as eosinophilic peroxidase (EPX) and eotaxin-3 (ET-3), may be of interest.^10^^,^^11^

The aim of the study was primarily to determine whether the NO system, as expressed by iNOS or NT, is more activated in EoE than in GERD patients compared with HVs. Secondarily, to compare the groups regarding the expression of EPX, ET-3, and Ga-3.

## METHODS

The material consisted of biopsies from the lower esophagus 2 cm above the z-line derived from each of the three categories (HVs, GERD patients, and EoE patients). These biopsies were saved from earlier studies.^12^^,^^13^ The ambition was to include 20 biopsies from each category. To allow for some expected ‘fall outs’, we randomly selected 72 cases from our biobank register (24 from each category). These were then randomized (https://stattrek.com/statistics/random-number-generator.aspx) and blinded with respect to category during later image analysis. Two biopsies from each group were kept from blinding for use as controls and for determining adequate dilutions of antibodies. Some biopsies, mainly in the HV category, could not be retrieved from the freezer; a few were damaged during the process of embedding, cutting, and staining; and some were of insufficient size to allow for all five cuts (five antibodies).

The GERD and EoE groups consisted of patients referred for esophagogastroduodenoscopy due to symptoms that were suggestive of EoE or GERD. These patients had to abstain from proton pump inhibitor (PPI) treatment for at least 2 weeks before the examination. Subjects were classified as suffering from GERD if they had typical symptoms, endoscopic and/or histopathologic esophagitis, and an eosinophil count of less than 15 eosinophils per high power field (hpf). If only microscopic esophagitis was observed, a pathologic 24-hour pH-metry was demanded. The diagnosis of EoE was assigned if subjects had a history of esophageal dysfunction and at least 15 eosinophils/hpf.

### The HV group

Consist of patients referred for surgery under general anesthesia who gave their consent to undergo esophagogastroscopy with biopsies (Table 1). The inclusion criteria were as follows: no ongoing or former symptoms affecting the upper gastrointestinal tract, no ongoing PPI or other acid-reducing treatment and no systemic disease.

**Table 1:** Healthy volunteers (*n* = 15)

	Age at biopsy	Men	ENT surgery	Elective laparascopic cholecystect.	Orthognat. corrective surgery		
Number		6	7	6	2		
Range	21–75						
Median	51						
Mean	50						
SD	15						
**GERD patients** (*n* = 20 EPX; *n* = 21 iNOS)							
	Age at biopsy	Men	NERD pH pos	GERD A	GERD B	GERD C	GERD D
Number		8	4	11	4	0	1
Range	25–71						
Median	51						
Mean	49						
SD	14						
**EoE patients** (*n* = 20)							
	Age at biopsy	Men	Eosinophils per HPF	Dysphagia score	EoEHSS stage	EoEHSS grade	
Number		18					
Range	18–80		15–150		0.0–0.9	0.1–0.9	
Median	42		31		0.41	0.20	
Mean	45		45		0.42	0.34	
SD	18		38		0.34	0.31	

After the exclusions mentioned above, 16 appropriate valid biopsies remained, of which one subject developed myeloma and was excluded because concomitant eosinophilia might occur. Among HVs, 15 images were available for analysis of all antibodies.

### The GERD group

Two biopsies were used as references, including tapering antibody dilutions. Another, in its EPX-stained version, escaped for some unclear reason, the automatic nightly digitalizing process (run as a single badge). One biopsy included only the epithelium of the gastric type and was excluded. For the GERD group, this left us with 20 images regarding EPX and 21 regarding the other antibodies.

#### The EoE group

Two biopsies were used as references, as described above. One biopsy could not be retrieved, and another escaped automatic digitalization, leaving us with 20 images for both EPX and iNOS and 19 for ET and Gal.

The antibodies used are:

- Monoclonal mouse-raised human EPX Antibody (EPO104) NBP2-32844-0.1 mg (www.novusbio.com/NBP2-32844),
- Monoclonal rabbit-raised human, mouse, rat iNOS (NOS2) antibody (K13-A) NBP1-33780-0.1 mL (www.novusbio.com/NBP1-33780),
- Monoclonal mouse-raised human NT antibody (EM-30) NBP1-96130, 0.1 mg (www.novusbio.com/NBP1-96130),
- Polyclonal rabbit-raised ET-3 (CCL26) antibody (https://www.avivasysbio.com/sd/tds/html_datasheet.php?sku=OACD05609),
- Monoclonal rat raised human, mouse galectin 3 antibody (eBioM3/38 [M3/38]), eBioscience.

The glasses were treated according to the two-step polymer method (EnVision FLEX, High pH, Dako Omnis), a visualizing system to detect primary mouse or rabbit antibodies through DAB+ Chromogen.

Approximately 280 IHC-stained object-glasses were photographed and used as high-resolution digitalized images for semiquantitative image analysis by a protocol with three scripts (see link below) in the opensource software QuPath v 0.4.2.^14^

In collaboration with coauthor Dr Chatterjee at The Centre for Cellular Imaging at the University of Gothenburg, a protocol for semi-quantifying the grade of DAB (3,3′-diaminobenzidine) stain was developed (https://www.protocols.io/blind/7EB76A466D8611EE8A230A58A9FEAC02). The results from the analysis are expressed both as the percentage of positive cells within a region of interest (ROI) and as the number of ‘positive’ cells per mm^2^ within this ROI. Cells with an ‘intensity’ (within a DAB color spectrum) exceeding a certain arbitrary limit are considered ‘positive’.

**Fig. 1:**
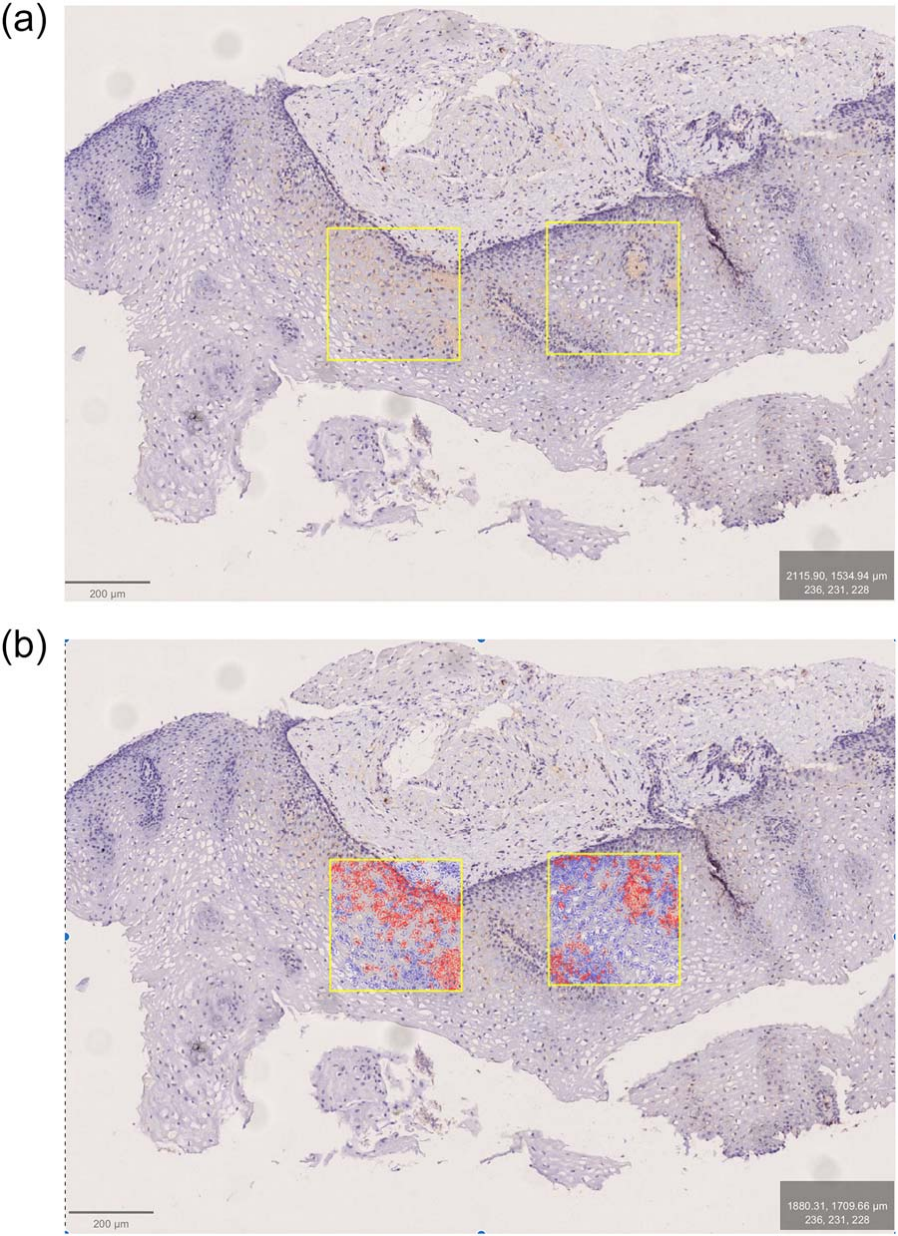
EoE patient. Antibody: eosinophilic peroxydase (EPX). The two most affected ROIs marked before (a) and after (b) positive cell detection.

**Fig. 2:**
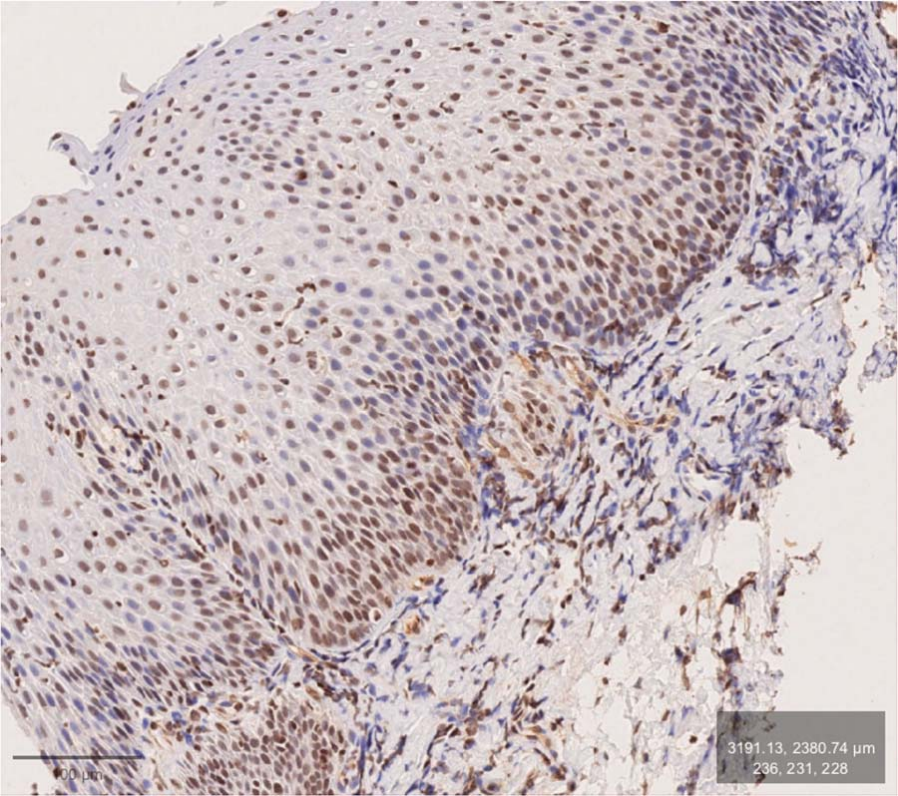
GERD patient. Antibody: inducible nitric oxide synthase (iNOS). Brownish DAB-stain of basal layers and adjoining submucosa. Distinct DIS (mainly in middle layers).

As EoE is considered a ‘patchy’ disease, we chose to count positive cells within an ROI consisting of a square with an area of 0.1 mm^2^ that could be placed over an area ‘by eye’ considered a ‘peak’ area showing maximal ‘brownish’ DAB staining (Figure 1 and 2). It should be placed to include all epithelial layers whenever possible. The size of the ROI was arbitrarily chosen to be small enough for localizing the area with ‘peak’ pathology and large enough to include representative parts of the epithelium. The ROI is approximately half the size of our high-power field, measuring 0.19–0.21 mm^2^. We similarly measured over the next most affected ROI. Hence, Tables 2–5 with suffix ‘a’ include two separate ROIs (‘peak’ and ‘subpeak’). Tables 2–5 with suffix ‘c’ represent the means of the two ROI readings.

### Statistics

For comparisons between groups, the Mann–Whitney U test for comparison of independent samples as two-tailed with a significance level of 5% was used.

The study was approved by the Regional Ethics Committee of Göteborg, Etikprövningsnämnden (EPN) (DNr 818-17).

## RESULTS

### Demographic data

The age and sex distribution (Table 1) did not differ significantly between HVs and GERD patients, while the mean age of EoE patients was approximately 5 years younger. Men were overrepresented in the EoE group. Most GERD patients were in LA Group A, representing relatively mild degrees of inflammation.

### Inducible nitric oxide synthase (iNOS)

#### Two separate ROIs per patient

A stepwise increase in mean values (MV) and % positive cells from HV via GERD to EoE was observed (Table 2a). A highly significant difference was observed between both GERD and EoE patients in comparison with HVs.

**Table 2a:** INOS, two ROIs per subject

**iNOS** Two ROIs per subject	Healthy volunteers		GERD patients		EoE patients	
	n 15 (30 ROIs)		n 21 (42 ROIs)		n 20 (40 ROIs)	
	Pos %	Nr Pos/mm2	Pos %	Nr Pos/mm2	Pos %	Nr Pos/mm2
Mean	38	2838	59	4619	63	4724
Mdn	36	1200	62	4040	65	3335
75th Perc	62	4880	86	7040	89	7665
25th Perc	10	335	35	2108	44	2280
IQR	50	4545	51	4933	45	5385
SD	31	3208	28	3087	29	3146
Comparison	HV vs. EoE		GERD vs. HV		EoE vs. GERD	
*P* (U-test)	**0.002**	**0.007**	**0.004**	**0.004**	ns	ns

#### Peak ROI values

The stepwise increase in MV between groups was confirmed, but the difference between GERD and EoE patients in comparison to HVs did not reach statistical significance (Table 2b).

**Table 2b:** INOS, one peak ROI per subject

**iNOS** One peak ROI per subject	Healthy volunteers		GERD patients		EoE patients	
	n 15		n 21		n 20	
	Pos %	Nr Pos/mm2	Pos %	Nr Pos/mm2	Pos %	Nr Pos/mm2
Mean	46	3611	65	5408	66	5349
Mdn	43	2440	65	4830	69	4555
75th Perc	76	5400	92	8170	91	8070
25th Perc	17	805	41	2210	49	2740
IQR	59	4595	51	5960	41	5330
SD	33	3475	27	3415	29	3341
Comparison	HV vs. EoE		GERD vs. HV		EoE vs. GERD	
*P* (U-test)	0.07	0.12	0.06	0,11	ns	ns

#### Mean ROI values

We confirmed the stepwise increase from HV via GERD to EoE (Table 2c). The difference observed above in Table 2a between each esophagitis group and HVs is slightly observable.

**Table 2c:** INOS, mean of 2 ROIs per subject

**iNOS** Mean of 2 ROIs per subject	Healthy volunteers		GERD patients		EoE patients	
	n 15		n 21		n 20	
	Pos %	Nr Pos/mm2	Pos %	Nr Pos/mm2	Pos %	Nr Pos/mm2
Mean	39	2838	59	4619	63	4724
Mdn	29	1285	59	4000	65	3780
75th Perc	62	4225	86	7385	87	7210
25th Perc	17	805	35	2045	49	2464
IQR	45	3420	51	5340	38	4746
SD	30	3068	27	2922	29	3043
Comparison	HV vs. EoE		GERD vs. HV		EoE vs. GERD	
*P* (U-test)	**0.03**	0.07	**0.04**	0.06	ns	ns

### Nitrotyrosine

The mean expression of NT seems to be most pronounced among GERD patients, with a significant difference compared to HVs. (Table 3a–3c).

**Table 3a:** 

**NT** Two ROIs per subject	Healthy volunteers		GERD patients		EoE patients	
	n 15 (30 ROIs)		n 21 (42 ROIs)		n 20 (40 ROIs)	
	Pos %	Nr Pos/mm2	Pos %	Nr Pos/mm2	Pos %	Nr Pos/mm2
Mean	35	1213	60	3306	46	2737
Mdn	27	670	66	2305	42	1755
75th Perc	64	1630	83	4953	84	4713
25th Perc	7	118	34	673	7	68
IQR	58	1512,5	49	4280	78	4645
SD	33	1694	31	3214	37	3075
Comparison	HV vs. EoE		GERD vs. HV		EoE vs. GERD	
*P* (U-test)	0.22	0.10	**0.003**	**0.001**	0.09	0.17

**Table 3b:** NT, one peak ROI per subject

**NT** One peak ROI per subject	Healthy volunteers		GERD patients		EoE patients	
	n 15		n 21		n 20	
	Pos %	Nr Pos/mm2	Pos %	Nr Pos/mm2	Pos %	Nr Pos/mm2
Mean	39	1667	63	3818	50	2948
Mdn	29	850	71	3180	45	2135
75th Perc	73	2495	85	5880	85	4820
25th Perc	9	275	41	1300	16	95
IQR	64	2220	44	4580	69	4725
SD	34	2181	30	3330	37	3257
Comparison	HV vs. EoE		GERD vs. HV		EoE vs. GERD	
*P* (U-test)	0.35	0.47	**0.047**	**0.02**	0.25	0.18

**Table 3c:** NT, mean of 2 ROIs per subject

**NT** Mean of 2 ROIs per subject	Healthy volunteers		GERD patients		EoE patients	
	n 15		n 21		n 20	
	Pos %	Nr Pos/mm2	Pos %	Nr Pos/mm2	Pos %	Nr Pos/mm2
Mean	34	1213	60	3313	46	2737
Mdn	23	495	67	2355	39	2013
75th Perc	64	2193	82	4125	85	4583
25th Perc	7	180	38	985	10	65
IQR	57	2013	44	3140	74	4518
SD	32	1312	31	3169	37	3092
Comparison	HV vs. EoE		GERD vs. HV		EoE vs. GERD	
*P* (U-test)	0.30	0.32	**0.02**	**0.02**	0.25	0.29

### Eosinophilic peroxidase (EPX)

#### Two separate ROIs per patient

Show a stepwise increase in mean values from HV via GERD to EoE (Table 4a). The number of positive detections (cells) was significantly higher among both EoE and GERD patients than among HV patients. The percentage of positive cells was significantly higher in EoE than in GERD.

**Table 4a:** EPX, Two ROIs per subject

**EPX** Two ROIs per subject	Healthy volunteers		GERD patients		EoE patients	
	n 15 (30 ROIs)		n 20 (40 ROIs)		n 20 (40 ROIs)	
	Pos %	Nr Pos/mm2	Pos %	Nr Pos/mm2	Pos %	Nr Pos/mm2
Mean	30	1475	25	2096	42	2700
Mdn	22	635	10	355	37	1910
75th Perc	53	1905	51	3078	69	3895
25th Perc	4	90	3	47,5	12	355
IQR	49	1815	48	3030	57	3540
SD	31	1812	31	3158	32	2618
Comparison	HV vs. EoE		GERD vs. HV		EoE vs. GERD	
*P* (U-test)	0.12	**0.03**	0.34	0.81	**0.01**	**0.03**

#### Peak ROI values

Confirm the stepwise increase in mean values between groups and show a significant difference comparing percent positive cells between HV and GERD (Table 4b).

**Table 4b:** EPX, One peak ROI per subject

**EPX** One peak ROI per subject	Healthy volunteers		GERD patients		EoE patients	
	n 15		n 20		n 20	
	Pos %	Nr Pos/mm2	Pos %	Nr Pos/mm2	Pos %	Nr Pos/mm2
Mean	35	1766	29	2576	46	3004
Mdn	28	860	14	570	45	2430
75th Perc	55	2375	54	5360	69	4273
25th Perc	5	155	5	97,5	15	685
IQR	72	2220	49	5263	54	3588
SD	33	2088	33	3563	33	2713
Comparison	HV vs. EoE		GERD vs. HV		EoE vs. GERD	
*P* (U-test)	0.3	ns	**0.01**	ns	ns	ns

#### Mean ROI values

Confirm the stepwise increase in means from HV via GERD to EoE, but no significance is reached in any parameter between the groups (Table 4c).

**Table 4c:** EPX, Mean of 2 ROIs per subject

**EPX** Mean of 2 ROIs per subject	Healthy volunteers		GERD patients		EoE patients	
	n 15		n 20		n 20	
	Pos %	Nr Pos/mm2	Pos %	Nr Pos/mm2	Pos %	Nr Pos/mm2
Mean	30	1475	25	2096	42	2700
Mdn	19	740	10	435	40	2035
75th Perc	48	1980	43	4040	68	4648
25th Perc	4	90	3	62,5	13	455
IQR	44	1890	40	3978	55	4193
SD	31	1780	31	3094	32	2574
Comparison	HV vs. EoE		GERD vs. HV		EoE vs. GERD	
*P* (U-test)	ns	ns	ns	ns	ns	ns

### Eotaxin-3

The mean values were generally higher in the two patient groups than in the HV group (Table 5a–5c). However, only by counting two ROIs per subject did these differences reach significance, while no significance was obtained when comparing the two patient groups to each other (Table 5a).

**Table 5a:** ET, Two ROIs per subject

**ET** Two ROIs per subject	Healthy volunteers		GERD patients		EoE patients	
	n 15 (30 ROIs)		n 21 (42 ROIs)		n 19 (38 ROIs)	
	Pos %	Nr Pos/mm2	Pos %	Nr Pos/mm2	Pos %	Nr Pos/mm2
Mean	10	740	29	2429	26	2121
Mdn	1	10	16	725	14	680
75th Perc	12	600	53	4563	50	2540
25th Perc	0	0	0	13	0	10
IQR	12	600	52	4550	50	2530
SD	16	1396	33	3140	29	3238
Comparison	HV vs. EoE		GERD vs. HV		EoE vs. GERD	
*P* (U-test)	**0.02**	**0.02**	**0.01**	**0.01**	ns	ns

**Table 5b:** ET, One peak ROI per subject

**ET** One peak ROI per subject	Healthy volunteers		GERD patients		EoE patients	
	n 15		n 21		n 19	
	Pos %	Nr Pos/mm2	Pos %	Nr Pos/mm2	Pos %	Nr Pos/mm2
Mean	12	835	32	2843	30	2498
Mdn	1	30	17	840	21	1290
75th Perc	21	925	62	4700	51	3275
25th Perc	0	5	1	40	3	25
IQR	21	920	61	4660	48	3250
SD	17	1467	34	3529	30	3483
Comparison	HV vs. EoE		GERD vs. HV		EoE vs. GERD	
*P* (U-test)	ns	0.05	ns	ns	ns	ns

**Table 5c:** ET, Mean of 2 ROIs per subject

**ET** Mean of 2 ROIs per subject	Healthy volunteers		GERD patients		EoE patients	
	n 15		n 21		n 19	
	Pos %	Nr Pos/mm2	Pos %	Nr Pos/mm2	Pos %	Nr Pos/mm2
Mean	10	740	27	2429	26	2121
Mdn	1	15	13	725	14	570
75th Perc	13	583	40	4180	43	2530
25th Perc	0	3	1	20	2	13
IQR	13	580	39	4160	41	2518
SD	15	1411	32	3091	29	3263
Comparison	HV vs. EoE		GERD vs. HV		EoE vs. GERD	
*P* (U-test)	ns	ns	ns	ns	ns	ns

### Galectin-3

Regarding Gal-3, no significant difference between groups was observed in any of the analyses (therefore not depicted).

## DISCUSSION

In accordance with our hypothesis, we found that iNOS was overexpressed in both EoE and GERD patients compared with that in HVs, but the expected difference between the two diseases was unsupported. There are few studies on the expression of NO via quantification of iNOS in esophageal biopsies, but in a small controlled study on active EoE patients, a significant overexpression of iNOS, which improved significantly after treatment, was found.^15^

Regarding NT, the mean values were higher in the disease groups than in the HVs. However, this overexpression of NT only reached statistical significance among GERD patients. Therefore, the suggestion of an even more pronounced NO activation among EoE patients in comparison to GERD patients is not supported by our results. Rather, GERD patients seem to stand out more than EoE patients regarding the overexpression of NT (Table 4a–4c). At first sight, this finding may be somewhat unexpected. It would, however, be plausible if GERD could be considered as a more constantly occurring inflammatory disease, while EoE is characterized as being a more short-lived relapsing inflammatory disease. This might reflect a more constant prevailing anatomic cause in GERD, while the grade of inflammation in EoE may vary by environment and diet. Furthermore, an overexpression of enzymatic NO production among GERD patients is in accordance with earlier human experimental controlled trials.^5^^,^^16^

Overall, our results are somewhat disappointing regarding the benefit of iNOS or NT as tools to differentiate between EoE and GERD.

Regarding EPX, a stepwise increase in the mean number of positive cell counts per mm^2^ was confirmed moving from HV, via GERD, to EoE. However, a pathognomonic association with EoE was not confirmed. This result conflicts with studies with similar IHC techniques.^17^^,^^18^ However, one of these studies was performed in children with eosinophilic gastritis and duodenitis, where the number of eosinophils counted is much higher.^17^ A controlled, but not randomized, study on EoE patients showed that EPX/mm2 correlated with the number of eos/hpf and could identify subjects with EoE.^19^ As these two last-mentioned studies, with a similar technique as ours, have obtained plausible results, there is reason to believe that the method itself should be able to work.

In the choice between eotaxin-1 or 3, we chose the latter, as it is reported to be more specific for epithelial cells than for smooth muscular cells.^19^ In our results regarding Eotoxin-3, the two disease states seem to differ somewhat from HVs, but the specificity for EoE over GERD is not supported. In another study, eotaxin was particularly useful for predicting responders.^20^ This result is opposed by a study configured in a similar way as ours but that conducted its analysis with two ‘blinded’ pathologists, which revealed a high specificity of EO-3 etaxin-3 for EoE.^21^ Therefore, perhaps it is difficult for the automatized systems to outperform the diagnostic ability of the experienced human eye. Then again, pathologists might be biased by other pathognomonic tissue appearances in EoE.

When this study was designed, galectin-3 was reported to be significantly upregulated in the EoE compared to the controls.^9^^,^^22^ We therefore included this tentative EoE marker, obviously without any significant findings. Based on today’s knowledge, galectin-10, a marker of regulatory eosinophils, would be a better choice.^23^

Among the strengths of our study is the automatized calculation using the same script with the same settings and within the same sized areas (ROIs) for the calculation of every image. A downside is the subjective placement. This problem is, however, counteracted by having the images randomized and blinded. Hence, the problem of subjective placing of ROIs should not add any systematic error, but subjective placing may increase the variability. Generally, the significance found in our study is more prominent when the double-number regions are counted. This finding suggests that the limited size in combination with considerable variability may imply a risk for type 2 error.

This study mainly confirms findings regarding iNOS and EPX and makes some noteworthy speculations regarding NT. However, it leaves clinicians without any new sharp diagnostic tools for differentiating and monitoring the two kinds of esophagitis.

## CONCLUSION

When measured as two separate ROIs in each histologic specimen, iNOS, NT, and ET-3 succeeded in differentiating between the healthy and inflammatory states. GERD patients displayed the highest levels of NT, differing significantly from HVs. Neither iNOS, nor NT alone, was able to differentiate between EoE and GERD. Therefore, Eosinophil Peroxidase is a better candidate. Our findings should be externally validated in new and larger patient groups.

## Notes

For communication with the editorial office: John Plate, MD, Department of Otorhinolaryngology, Head and Neck Surgery, NU-Hospital Group, NAL Hospital, Larketorpsvagen 20, 461 73 Trollhattan, Sweden. Email workplace: John.Plate@vgregion.se, E-mail private: Plate_93@hotmail.com, Telephone workplace (exchange): +46 10 435 00 00, Telephone private: +46 70 433 99 89.

Specific author contributions: John Plate (Data curation, Formal analysis, Validation, Writing—review & editing), Mogens Bove (Conceptualization, Data curation, Formal analysis, Methodology, Supervision, Validation, Visualization, Writing—original draft, Writing—review & editing), Helen M. Larsson (Investigation, Supervision, Writing—review & editing), Elisabeth Norder Grusell (Investigation, Validation, Writing—review & editing), Nabanita Chatterjee (Methodology, Software, Analysis, Validation, Visualization, Writing—review & editing), Leif E. Johansson (Validation, Writing—review & editing), and Henrik Bergquist (Conceptualization, Resources, Project administration, Supervision, Visualization, Writing—review & editing)
